# Supplementary material for: Circulation of pertussis and poor protection against diphtheria among middle-aged adults in 18 European countries
Source: Nat Commun. 2021 May 17;12:2871. doi: 10.1038/s41467-021-23114-y (PMC8128873; doi:10.1038/s41467-021-23114-y)
Supplement: Supplementary file 1 — Supplementary information [file 41467_2021_23114_MOESM1_ESM.pdf]

Supplementary Information to the manuscript

Circulation of pertussis and poor protection against diphtheria among middle-aged adults in 18 European countries

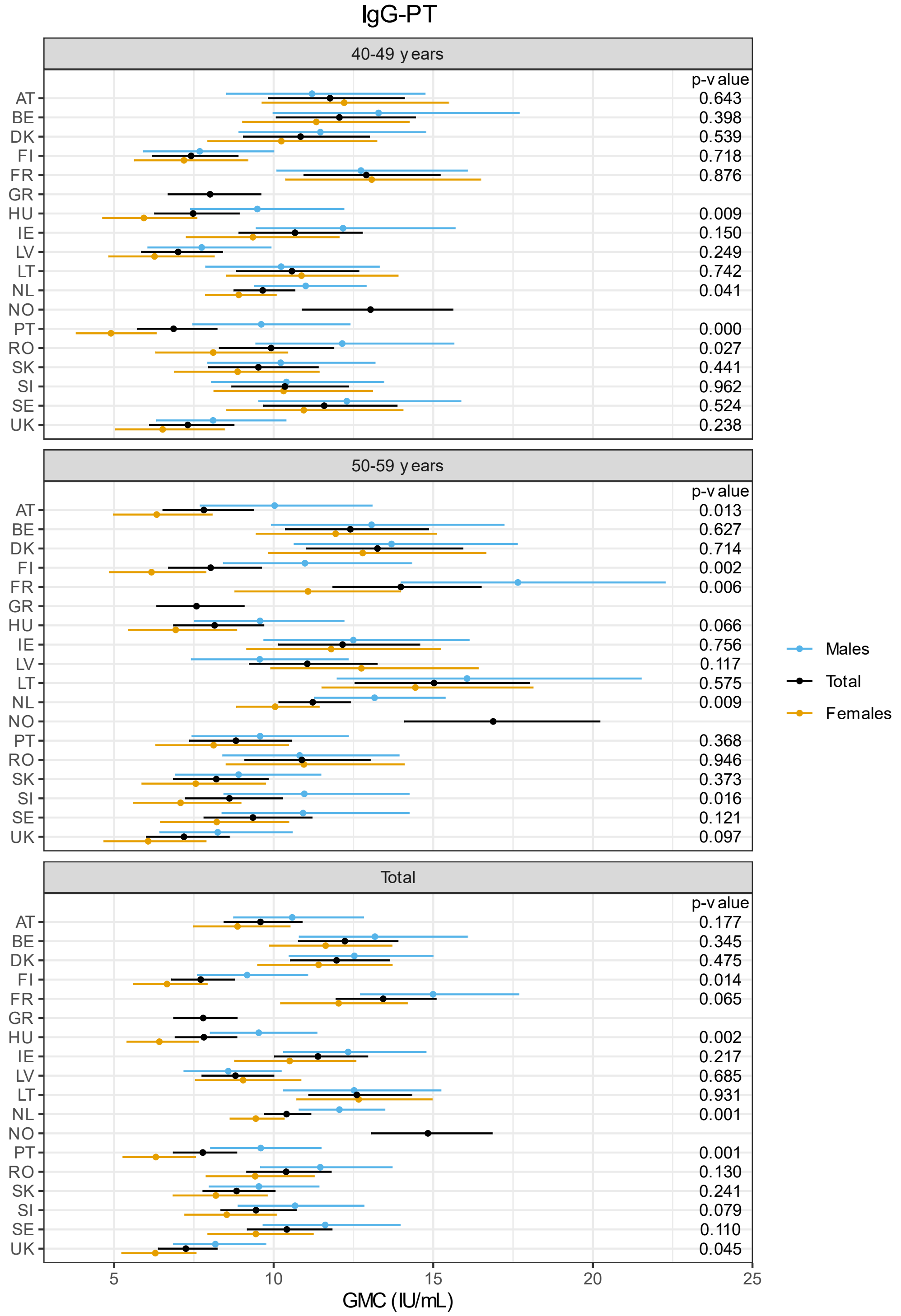

Supplementary Fig.1 GMCs for IgG-PT in IU/mL in the two age groups (40-49 and 50-59) separately and in the total cohort, and subdivided by sex per country (Y-axis). The dots indicate the estimated geometric mean, the bars the corresponding 95% confidence intervals. The estimates and *p* values of the differences are obtained by a linear regression model. Abbreviations of all participating countries and the number of samples included in the study are listed in Table 1.

Supplementary Table 1 ***p*** **values for the differences in seroprevalences between the age groups, sex and countries**

Antibody level	Age	Sex	Country
IgG-PT ≥100 IU/ml	p=0.846	p=0.802	p=0.023
IgG-PT 50 to <100 IU/ml	p=0.212	p=0.082	p=0.007
IgG-PT ≥50 IU/ml	p=0.038	p=0.020	p<0.001
IgG-Dt <0.01 IU/ml	p<0.001	p<0.001	p<0.001
IgG-Dt <0.1 IU/ml	p<0.001	p<0.001	p<0.001
IgG-TT <0.01 IU/ml	p=0.902	p=0.986	p=0.491
IgG-TT <0.1 IU/ml	p=0.001	p<0.001	p<0.001

p values were analysed using the likelihood ratio test on a binomial generalised linear regression model.

Supplementary Table 2 **Notification rates (N/100,000) for subjects ≥15y from the ECDC Surveillance Atlas for pertussis during 2015-2018 by country.**

	Notification Rate (N/100,000), all cases, age 15 and above				Collection time
**Country**	**2015**	**2016**	**2017**	**2018**	
Austria (AT)	5.52	10.20	11.23	16.00	2015-2016
Belgium (BE)	–	–	–	–	2017
Denmark (DK)	10.56	23.34	12.62	12.16	2015-2016
Finland (FI)	1.64	4.94	4.32	5.82	2015-2016
France (FR)	–	–	–	–	2015-2016
Greece (GR)	0.01	0.01	0.00	0.02	2015
Hungary (HU)	0.04	0.02	0.16	0.19	2017
Ireland (IE)	0.92	1.91	2.86	1.12	2016-2017
Latvia (LV)	5.69	10.07	3.40	5.34	2015-2016
Lithuania (LT)	0.56	0.53	0.37	0.42	2016
Netherlands (NL)	27.97	22.89	21.00	19.58	2016-2017
Norway (NO)	32.88	34.81	35.60	33.70	2015-2016
Portugal (PT)	0.28	0.75	0.28	0.08	2015-2016
Romania (RO)	0.09	0.05	0.10	0.06	2018
Slovak Republic (SK)	6.77	5.57	3.61	7.12	2016-2018
Slovenia (SI)	1.42	3.64	6.26	5.81	2015-2016
Sweden (SE)	4.89	5.31	6.27	5.55	2016
United Kingdom (UK)	8.01	10.99	6.80	5.18	2015-2016

Available at: https://ecdc.europa.eu/en/pertussis/surveillance-and-disease-data/atlas
