# Supplementary material for: Circulation of pertussis and poor protection against diphtheria among middle-aged adults in 18 European countries
Source: Nat Commun. 2021 May 17;12:2871. doi: 10.1038/s41467-021-23114-y (PMC8128873; doi:10.1038/s41467-021-23114-y)
Supplement: Supplementary file 3 — Reporting Summary [file 41467_2021_23114_MOESM3_ESM.pdf]

nature research

Corresponding author(s): Guy AM Berbers, Qiushui He

Last updated by author(s): Mar 17, 2021

Reporting Summary

Nature Research wishes to improve the reproducibility of the work that we publish. This form provides structure for consistency and transparency in reporting. For further information on Nature Research policies, see our Editorial Policies and the Editorial Policy Checklist.

Statistics

For all statistical analyses, confirm that the following items are present in the figure legend, table legend, main text, or Methods section.

n/a	Confirmed	
☐	☒	The exact sample size (*n*) for each experimental group/condition, given as a discrete number and unit of measurement
☐	☒	A statement on whether measurements were taken from distinct samples or whether the same sample was measured repeatedly
☐	☒	The statistical test(s) used AND whether they are one- or two-sided<br>*Only common tests should be described solely by name; describe more complex techniques in the Methods section.*
☐	☒	A description of all covariates tested
☐	☒	A description of any assumptions or corrections, such as tests of normality and adjustment for multiple comparisons
☐	☒	A full description of the statistical parameters including central tendency (e.g. means) or other basic estimates (e.g. regression coefficient) AND variation (e.g. standard deviation) or associated estimates of uncertainty (e.g. confidence intervals)
☐	☒	For null hypothesis testing, the test statistic (e.g. *F*, *t*, *r*) with confidence intervals, effect sizes, degrees of freedom and *P* value noted<br>*Give P values as exact values whenever suitable.*
☒	☐	For Bayesian analysis, information on the choice of priors and Markov chain Monte Carlo settings
☒	☐	For hierarchical and complex designs, identification of the appropriate level for tests and full reporting of outcomes
☒	☐	Estimates of effect sizes (e.g. Cohen's *d*, Pearson's *r*), indicating how they were calculated

Our web collection on statistics for biologists contains articles on many of the points above.

Software and code

Policy information about availability of computer code

Data collection	IgG antibody levels against pertussis toxin, diphtheria toxoid and tetanus toxin in all serological samples were measured using the Bioplex LX200 and the software programme Bioplex Manager 6.2 (Bio-Rad Laboratories, Hercules, CA, USA). Further data collection has been done in Excel (Microsoft Office 365, version 16.0)
Data analysis	The analysis has been done using R 4.0.2. This is open-source software. The R code of the statisticus involved, Dr. Jan van de Kassteele, can be found on his GitHub page. See https://github.com/kassteele/EU_Pertussis_seroprevalence

For manuscripts utilizing custom algorithms or software that are central to the research but not yet described in published literature, software must be made available to editors and reviewers. We strongly encourage code deposition in a community repository (e.g. GitHub). See the Nature Research guidelines for submitting code & software for further information.

Data

Policy information about availability of data

All manuscripts must include a data availability statement. This statement should provide the following information, where applicable:

- Accession codes, unique identifiers, or web links for publicly available datasets
- A list of figures that have associated raw data
- A description of any restrictions on data availability

The authors declare that all relevant data supporting the findings of this study are available within the paper [and its supplementary information files]. The original raw data from all serological measurements that support the findings of this study are available from the corresponding author upon reasonable request.

Field-specific reporting

Please select the one below that is the best fit for your research. If you are not sure, read the appropriate sections before making your selection.

- [x] Life sciences
- [ ] Behavioural & social sciences
- [ ] Ecological, evolutionary & environmental sciences

For a reference copy of the document with all sections, see nature.com/documents/nr-reporting-summary-flat.pdf

Life sciences study design

All studies must disclose on these points even when the disclosure is negative.

Sample size	The initial sample size for a proportion in a simple random sample is calculated as (Cochrane, Cochran, Willian G. (1977) Sampling Techniques, 3rd Edition. John Wiley & Sons): n0 = (z2/α/2)x p(1-p)/d2, where zα/2 is the value for a standard normal distribution at confidence level α/2; p is the proportion of population with pertussis infections in the last two years; and d is the precision of the estimate. For example, with 1 −α = 0.95 (zα/2=1.96), p = 0.10, and d = 0.05, the initial sample size is n0 = 138.3 individuals; if instead p = 0.20 with the same precision and confidence level, the sample size is 245.9. (z2 means z square , and d2 also d square; n, z, p and d are in italic in the formula).
Data exclusions	No data were excluded.
Replication	Serum samples were measured in two dilutions (1/200 and 1/4,000), with in-house references calibrated against international standards, control sera and blanks included on each plate and MFI was converted to IU/mL by interpolation from a five-parameter logistic standard curve. As control for possible drift of the assay in time and different bead batches used, 5% of randomly selected samples per country were assessed in an extra duplicate measurement (see materials and methods). All duplicates were acceptable and met the criteria as described in the SOP of the method.
Randomization	Samples from participants were not allocated in specific groups. The sample results were compared statistically by country origin, by age and by sex as described in materials and methods.
Blinding	The laboratory technicians were blinded during the serological measurements for the samples origin.

Reporting for specific materials, systems and methods

We require information from authors about some types of materials, experimental systems and methods used in many studies. Here, indicate whether each material, system or method listed is relevant to your study. If you are not sure if a list item applies to your research, read the appropriate section before selecting a response.

Materials & experimental systems

n/a	Involved in the study	
[ ]	[x]	Antibodies
[x]	[ ]	Eukaryotic cell lines
[x]	[ ]	Palaeontology and archaeology
[x]	[ ]	Animals and other organisms
[ ]	[x]	Human research participants
[x]	[ ]	Clinical data
[x]	[ ]	Dual use research of concern

Methods

n/a	Involved in the study	
[x]	[ ]	ChIP-seq
[x]	[ ]	Flow cytometry
[x]	[ ]	MRI-based neuroimaging

Antibodies

Antibodies used	For the multiplex serological analyses we used:<br>1. Secondary antibody: R-Pphycoerythrin-conjugated AffiniPure F(ab')$_2$ Fragment Goat anti-Human IgG, Fcγ fragment specific (minimal cross-reaction to Bovine, Horse and Mouse Serum Proteins, Code Number 109-116-098, Jackson Immunoresearch, West Grove, PA, USA),<br>2. In-house Pertussis Luminex standard calibrated against the WHO International Standard Pertussis Anti-serum (Human) 1st IS NIBSC code 06/140 (in IU/ml),<br>3. In-house Diphtheria Luminex standard serum values assigned in IU/ml calibrated against the International standard for diphtheria (NIBSC code DI, equine),<br>4. In-house Tetanus Luminex standard serum values assigned in IU/ml calibrated against the International standard for tetanus (NIBSC code TE-3, human). NIBSC in Blanche Lane, South Mimms, Potters Bar, Hertfordshire, EN6 3QG (UK).
Validation	Validation of these antibodies has been described on the manufacturer's websites.<br>Validation of the multiplex immuno assay (MIA) has been described in several earlier publications and is referred to in the manuscript.

Human research participants

Policy information about studies involving human research participants

Population characteristics	The covariant relevant population characteristics of the participants were age, sex and country/location. Samples were anonymised.
Recruitment	The study design was a random sample of retrospective collections of anonymised leftover serum samples or anonymised samples from nationwide serosurveillance studies with formal approval from a medical ethical committee during 2015-2018. The locations per country are described in Table 1. Recruitment of the nation-wide serosurveillance study in the Netherlands is described in Verberk JDM, Vos RA, Mollema L, van Vliet J, van Weert JWM, de Melker HE, van der Klis FRM. Third national biobank for population-based seroprevalence studies in the Netherlands, including the Caribbean Netherlands. BMC Infect Dis. 2019 May 28;19(1):470. doi: 10.1186/s12879-019-4019-y.
Ethics oversight	Ethical approval was obtained from the Medical Ethics Committee Noord-Holland, the Netherlands (Pienter3 study: NL5467 (NTR5611)). For the use of the anonymised left-over samples from the other countries no ethical approval was required.

Note that full information on the approval of the study protocol must also be provided in the manuscript.
